# Reference Values for Myocardial Strain by Cardiac Magnetic Resonance Feature Tracking: Insights From Healthy Volunteers and Heart Failure Patients Using Caas MR

**DOI:** 10.31083/RCM41521

**Published:** 2026-01-08

**Authors:** Karl Jakob Weiss, Shing Ching, Patrick Doeblin, Irene Carrión-Sánchez, Karina Carrizosa, Radu Tanacli, Stefanie Werhahn, Jana Veit, Rebecca Elisabeth Beyer, Nicole Mittmann, Christian Stehning, Gaston Vogel, Hans-Dirk Düngen, Moritz Blum, Djawid Hashemi, Sebastian Kelle

**Affiliations:** ^1^Department of Cardiology, Angiology and Intensive Care Medicine, Deutsches Herzzentrum der Charité, 13353 Berlin, Germany; ^2^Charité – Universitätsmedizin Berlin, Freie Universität Berlin and Humboldt-Universität zu Berlin, 10117 Berlin, Germany; ^3^DZHK (German Centre for Cardiovascular Research), Partner Site Berlin, 10785 Berlin, Germany; ^4^Division of Cardiology, Department of Medicine and Geriatrics, United Christian Hospital, Hong Kong, China; ^5^Division of Cardiovascular Imaging, Department of Cardiology, University Hospital Ramón y Cajal, 28034 Madrid, Spain; ^6^Philips Clinical Science, Philips Healthcare, 22335 Hamburg, Germany; ^7^Pie Medical Imaging BV, Product Management, 6227 AK Maastricht, The Netherlands; ^8^BIH Biomedical Innovation Academy, BIH, Berlin Institute of Health at Charité – Universitätsmedizin Berlin, 10117 Berlin, Germany

**Keywords:** cardiac magnetic resonance, strain, feature tracking, deformation imaging, healthy, heart failure, patients

## Abstract

**Background:**

Magnetic resonance imaging (MRI) allows for the assessment of myocardial strain and identification of heart failure (HF) patients with reduced (HFrEF), mildly reduced (HFmrEF), or preserved (HFpEF) left ventricular ejection fraction (LVEF). The cardiovascular angiographic analysis system magnetic resonance (Caas MR) strain (Pie Medical Imaging) has recently been implemented in the IntelliSpace Portal Suite (Philips Healthcare) to assess the global longitudinal strain (GLS), global circumferential strain (GCS), and global radial strain (GRS). However, standard values for this software across different HF entities, as well as normal values, have yet to be established. Thus, this study aimed to establish reference values for the GLS, GCS, and GRS using the Caas MR strain in healthy individuals and HF patients, to assess the ability of these parameters to differentiate between HF subtypes, and to compare CAAS-derived strain values with those obtained using CVI42 software.

**Methods:**

Using a 1.5 T Philips Achieva scanner, we analyzed 19 healthy volunteers and 56 HF patients (HFpEF, n = 19; HFmrEF, n = 20; and HFrEF, n = 17) using the feature tracking post-processing software Caas MR Strain. GLS, GCS, and GRS were quantified using 4-chamber-view, 2-chamber-view, and short-axis (SAX) cine images. All volunteers and patients were evaluated by CVI42 to analyze inter-vendor reliability with a validated software.

**Results:**

Mean GLS, GCS, and GRS by Caas MR Strain were significantly different for healthy volunteers compared to HF patients (GLS –15.8 ± 1.9% vs. –11.7 ± 3.0%, *p* < 0.001; GCS –17.0 ± 2.6% vs. –11.4 ± 3.3%, *p* < 0.001; GRS 27.3 ± 6.2% vs. 14.5 ± 5.5%, *p* < 0.001). The upper limit of the 99% confidence interval for healthy volunteers was –14.6% for GLS, –15.3% for GCS and the lower limit of the 99% CI for GRS was 23.1%. GLS, GRS, and GCS by Caas MR Strain were significantly different among HF entities (*p* < 0.001). Intervendor comparison showed very good agreement for GLS and GRS between Caas MR Strain and CVI42 (GLS *r* = 0.86, *p* < 0.001; GCS *r* = 0.83, *p* < 0.001; GRS *r* = 0.76, *p* < 0.001).

**Conclusion:**

Magnetic resonance imaging assessment of left ventricular myocardial strain using Caas MR Strain software reliably identifies HF patients. Discrimination between the different HF entities is potentially feasible by GLS, GCS, and GRS. Intervendor agreement was most robust for GLS and GCS, but less robust for GRS. For practical clinical use, we propose cut-off values for GLS above –15%, GCS above –15%, and GRS below 23% to define pathological findings.

## 1. Introduction

Heart failure (HF) is a complex cardiovascular disorder characterized by impaired cardiac function and increased morbidity and mortality worldwide [1, 2, 3]. Accurate assessment of myocardial deformation in human subjects has emerged as a valuable tool in evaluating cardiac function and identifying early abnormalities in patients with HF [2, 4, 5, 6].

Myocardial strain assessment by cardiac magnetic resonance (CMR) imaging is a useful tool to measure global and regional myocardial function and deformation quantitatively in CMR imaging [5]. It has been shown to offer insights regarding the patient’s prognosis, both in acute and chronic HF [6, 7, 8, 9, 10]. Left ventricular deformation can be quantified in three dimensions: longitudinal strain, circumferential strain, and radial strain [11]. Among the various imaging techniques available, feature tracking (FT-MRI) strain analysis has gained significant attention due to its ability to quantify myocardial deformation using standard cine CMR sequences [5, 12]. After manual or automated segmentation of the myocardium in end-diastole, tracking of distinct pixel patterns (around 15 mm^2^) throughout the cardiac cycle for the entire myocardium reveals the myocardium deformation field from which the strain in three main axes are computed: longitudinal, radial, and circumferential strain [13]. Previous studies have demonstrated the potential of FT-MRI strain analysis in discriminating between healthy individuals and patients with HF [14, 15, 16]. This technique has been proven to be as reliable as acquisition-based strain-measurements. Post-processing solutions that allow for FT-MRI strain analysis based on standard steady-state free precession (SSFP) sequences are now offered by many vendors [17, 18]. However, universal standards for the interpretation of FT-MRI results do not currently exist, and different vendors and methods for analysis can significantly affect deformation values [18, 19, 20].

The cardiovascular angiographic analysis system magnetic resonance (Caas MR) Strain (Pie Medical Imaging) has recently been implemented in the IntelliSpace Portal Suite (Philips Medical Systems Nederland B.B., Best, The Netherlands) to assess global longitudinal (GLS), circumferential (GCS), and radial strain (GRS). It relies only on a two-chamber (2CH) and a four-chamber (4CH) view to calculate GLS and uses a full stack short axis sequence to assess GCS and GRS. However, normal values for these measurements have not yet been established.

Although previous publications have reported myocardial strain parameters using the present patient cohort, this study provides novel insights by establishing reference values for GLS, GCS and GRS using the Caas MR Strain software and reporting the first direct comparison with the widely used CVI42 platform. This study also proposes potentially clinically applicable cut-off values for pathological strain patterns in HF patients, aiming to support decision-making in routine practice. The aims of this study were threefold: to establish cutoff values for GLS, GCS, and GRS that distinguish healthy volunteers from HF patients, to evaluate the discriminatory power of these parameters in different HF subgroups, and to validate the comparability and agreement between software solutions from different vendors.

## 2. Material and Methods

### 2.1 Study Population

This study was performed at two centers in Berlin, Germany, the Charité–University Medicine Berlin and the German Heart Centre Berlin, in the years 2017 and 2018. Data from this cohort have been previously published by our research group in studies addressing related aspects of HF phenotyping and tissue characterization [14, 15, 21]. We included previously reported demographic data to support the current original strain analysis using Caas MR Strain. We prospectively identified 19 healthy volunteers without cardiovascular comorbidities or regional wall motion abnormalities and 56 HF patients, presenting with symptoms of HF and an increased N-terminal pro b-type natriuretic peptide (NT-proBNP) (>220 pg/mL). In the HF group, 17 patients were classified as HF with reduced left ventricular ejection fraction (HFrEF), defined as a left ventricular ejection fraction (LVEF) of <40%, 20 patients had HF with mid-range left ventricular ejection fraction (HFmrEF, LVEF 40–49%), and 19 patients had HF with preserved left ventricular ejection fraction (HFpEF, LVEF ≥50%) [22]. All participants were prospectively recruited and had taken part in previous studies conducted by our group [14, 15, 21]. Exclusion criteria for the healthy volunteers were known cardiovascular risk factors, any pre-existing diseases or medications, impaired LVEF (<55%), or pathological findings on a 12-lead electrocardiogram (ECG) or CMR. In all the participants, incomplete CMR data for feature tracking analysis led to their exclusion. The study protocol was in full agreement with the Declaration of Helsinki. All studies were reviewed and approved by the Charité–Universitätsmedizin Berlin Ethics Committee, which complied with the Declaration of Helsinki and were registered at the German Register for Clinical Studies (DRKS) (registration number: DRKS00015615).

### 2.2 CMR Protocol

After informed consent was obtained in all HF patients and volunteers, CMR was performed on a 1.5 Tesla (T) CMR scanner (Achieva, Philips Healthcare, Best, The Netherlands) using a five-element phased array receiver coil. Patients and volunteers were placed in the supine position, and images were acquired during breath holds of 10 to 15 seconds by using vector electrocardiogram gating. A rapid balanced SSFP sequence with a repetition time (TR) = 3.3 ms, echo time (TE) = 1.6 ms, flip angle = 60°, voxel size = 1.8 × 1.7 × 8.0 mm^3^, twofold SENSE acceleration, and 50 phases per cardiac cycle was used to acquire cine images covering the entire left ventricular myocardium. Cine images were acquired with multiple breath holds in two-chamber (2CH), three-chamber (3CH), and four-chamber (4CH) planes. A full stack of short-axis (SAX) slices covering the entire left ventricle (LV) was acquired, with a TR = 2.9 ms, TE = 1.4 ms, flip angle = 60°, voxel size = 1.63 × 1.63 × 8.0 mm^3^ and 25 phases per cardiac cycle. Three SAX slices in the basal, midventricular, and apical planes were acquired, using the same imaging parameters as for the long-axis planes.

### 2.3 Image Analysis

**Fig. 1.**
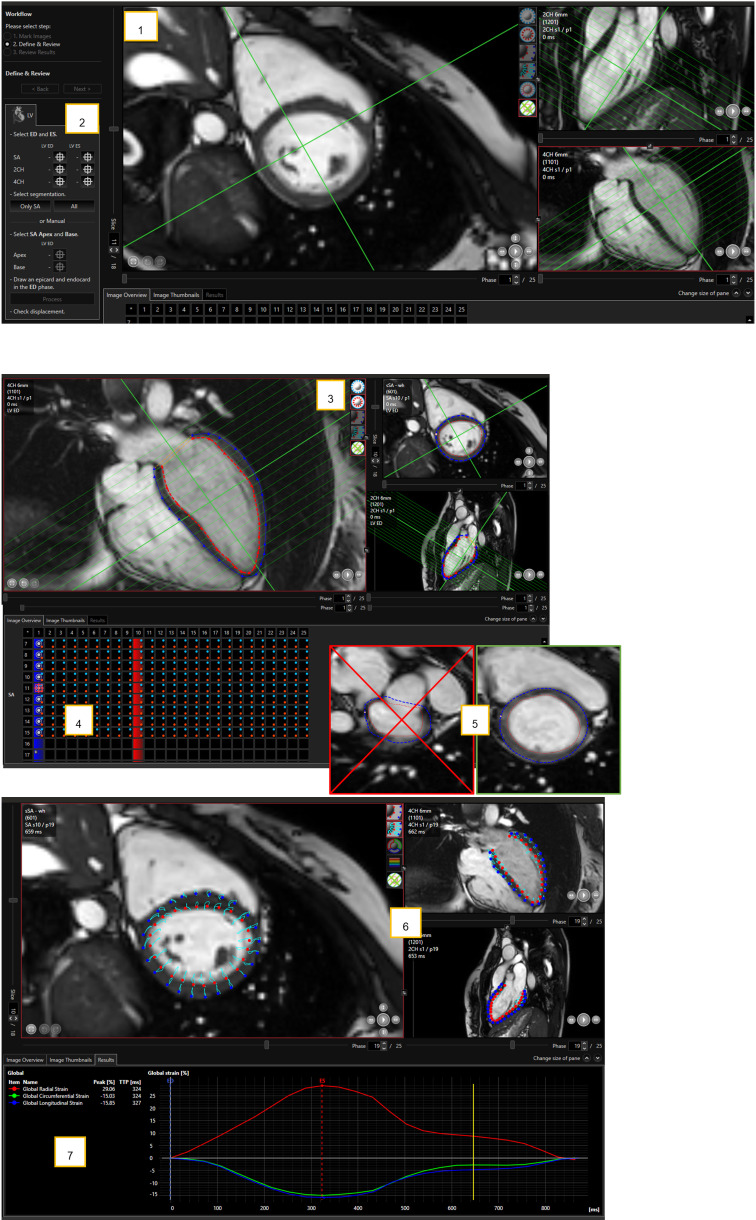
**Semi-automatic left ventricular segmentation and strain analysis using Caas MR Strain**. (1,2) Example of semi-automatic segmentation by Caas MR Strain. (1) Short-axis (SAX, left) and long-axis (2CH and 4CH, right) SSFP cine images were loaded directly from Philips Intellispace to the Caas MR Strain. The green lines indicate the intersection lines in the short and long axis. (2) Users can choose between automatic segmentation or manually define endo- and epicardial contours. Image processing was performed using Caas MR Strain software (Pie Medical Imaging, Maastricht, The Netherlands). (3–5) Example of a semi-automatic strain analysis by Caas MR Strain. (3) After automatic segmentation, endo- (red dotted line) and epicardial (blue dotted line) borders can be visually assessed and corrected if necessary. (4) The basal and apical slice for the short axis (SAX) stack can be defined manually. (5) SAX slices in the diastolic or systolic phases containing the left ventricular outflow tract (LVOT) should be excluded from the analysis. Caas MR Strain, Pie Medical Imaging, Maastricht, The Netherlands. (6,7) Example of a semi-automatic strain analysis by Caas MR Strain. (6) Deformation is automatically derived from feature tracking which can be assessed visually (light blue lines), for endo- (red dotted line) and epicardial (blue dotted line) borders. (7) Global strain values (not shown) and segmental values are given for user defined segments (not shown) or the American Heart Association (AHA) 17-segment model. Caas MR, cardiovascular angiographic analysis system magnetic resonance; 2CH, two-chamber; 4CH, four-chamber; SSFP, standard steady-state free precession.

All image analyses were performed according to consensus recommendations [23]. After careful scanning for artifacts independently by two experienced readers (European Association of Cardiovascular Imaging (EACVI) level III), cine images were analyzed using commercially available software (IntelliSpace Portal V 12.1, Philips Medical Systems Nederland BV, Best, The Netherlands; Caas MR Strain, Pie Medical Imaging, Maastricht, The Netherlands). All volunteers and patients were further analyzed by CVI42 (CVI42 version 5.13.7, Circle Cardiovascular Imaging Inc., Calgary, Alberta, Canada). Caas MR Strain uses feature tracking to detect the ventricular deformation patterns. Feature tracking is based on a block-matching approach. It first identifies anatomic features in the CMR image along the myocardial boundaries, then defines regions of interest around these locations and finally tracks them along the cardiac cycle by looking for the most similar region as illustrated in Fig. 1. The strain algorithm in Caas MR Solutions represents the strain values of the whole myocardium. The type of strain calculated is the Lagrangian strain.

LV function and volumes were quantified in a whole SAX stack according to the recommendation of the Society for Cardiovascular Magnetic Resonance (SCMR) with papillary muscles excluded from the LV volume for both vendors [23]. In the strain analysis, if the left ventricular outflow tract (LVOT) was seen in diastolic and/or systolic phases, SAX slices were completely excluded. Endo- and epicardial contours were automatically drawn in the end-diastolic and end-systolic phases, individually checked, and manually adjusted if necessary. The entire SAX was used to evaluate both segmental and global circumferential (GCS) and radial strain (GRS) by Caas MR Strain and CVI42. 2CH and 4CH were used by Caas MR Strain and 2CH, 3CH, and 4CH by CVI42 to assess segmental and global longitudinal strain (GLS). Left ventricular segmentation was based on the 17-segment model from the American Heart Association (AHA) excluding the apex (Segment 17) [24]. An example of an illustration of a strain analysis by Caas MR Strain is shown in Fig. 1.

### 2.4 Statistical Analysis

Continuous variables are presented as mean ± standard deviation (SD), categorical variables are expressed as numbers and proportions. Patient and CMR characteristics were compared using the Mann–Whitney U test or Kruskal Wallis test for continuous variables and the chi-square or Fisher’s exact test for categorical and ordinal variables. Normal global strain values were calculated as the upper or lower limit of the 99% confidence interval (CI), given as mean ± 2.576 SD [13]. The cut-off points were obtained using the upper limit of the 95% confidence intervals of the mean values for healthy volunteers for GLS and for GCS and using the lower limit of the 99% confidence interval for GRS. Inter-rater reliability of strain values between the two readers was assessed by intra-class correlation (ICC) using a two-way random model. Inter-vendor agreement was evaluated using Pearson’s correlation coefficient and Bland-Altman analysis to identify systematic bias. Statistical significance was defined at *p* < 0.05. Pairwise comparisons following KruskalWallis testing were conducted using Dunn’s test with Bonferroni adjustment to control the familywise error rate. Statistical analysis was performed using IBM SPSS Statistics 29.0 (IBM Corp., Armonk, NY, USA).

## 3. Results

### 3.1 Demographics

We identified 19 healthy volunteers without cardiovascular comorbidities or regional wall motion abnormalities and 56 HF patients [a total of 75 participants, 27 female (36%), mean age 68 ± 11 years, mean BMI 27 ± 4 kg/m^2^] who underwent a cardiac MRI between 06/04/2017 and 19/11/2018. HF patients were significantly older than healthy volunteers (mean age 69 ± 11 vs. 63 ± 9 years, *p* = 0.024), had higher left ventricular end-diastolic and end-systolic volumes (LVEDV, mL; LVEDVi, mL/m^2^; LVESV, mL; LVESVi, mL/m^2^) and left ventricular mass (LVM, g; LVMi, g/m^2^) than healthy volunteers. Left ventricular ejection fraction (LVEF, %) and indexed left ventricular stroke volume (SVi, mL/m^2^) were significantly lower in HF patients. Detailed values of HF patients are shown in Table 1 (Ref. [14, 15, 21]) and have been previously published [14, 15].

**Table 1.** **Baseline characteristics of healthy volunteers and heart failure patients**.

	Healthy volunteers (n = 19)	HF patients (n = 56)	Test (statistic)	*p* value
Female, n (%)	9 (47)	18 (32)	χ^2^ = 1.43	0.232
Age (years)	63 ± 9	69 ± 11	*t* = 2.31	0.024*
Height (cm)	171 ± 11	170 ± 8	*t* = –0.17	0.868
Weight (kg)	74 ± 14	80 ± 13	*t* = 1.62	0.109
BMI (kg/m^2^)	25 ± 4	28 ± 4	*t* = 1.98	0.051
BSA (m^2^)	2 ± 0	2 ± 0	*t* = 1.11	0.269
LVEDV (mL)	144 ± 36	192 ± 69	*t* = 2.87	0.005*
LVEDVi (mL/m^2^)	76 ± 14	98 ± 31	*t* = 2.85	0.006*
LVESV (mL)	53 ± 19	110 ± 61	*t* = 3.97	<0.001
LVESVi (mL/m^2^)	28 ± 9	55 ± 28	*t* = 3.96	<0.001*
LVM (g)	85 ± 30	115 ± 40	*t* = 2.98	0.004*
LVMi (g/m^2^)	44 ± 12	59 ± 18	*t* = 3.40	0.001*
LVEF (%)	64 ± 6	46 ± 12	*t* = –6.08	<0.001*
SV (mL)	91 ± 19	82 ± 16	*t* = –1.90	0.062
SVi (mL/m^2^)	48 ± 7	43 ± 7	*t* = –2.77	0.007*

Values are presented as n (%) for categorical variables and as mean ± standard deviation for continuous variables. Statistically significant comparisons (*p* < 0.05) are indicated with an asterisk (*). BMI, body mass index; BSA, body surface area (DuBois); LVEDV/LVEDVi, left ventricular end-diastolic volume and -index; LVESV/LVESVi, left ventricular end-systolic volume and -index; LVM/LVMi, left ventricular mass and -index; LVEF, left ventricular ejection fraction; SV/SVi, left ventricular stroke volume and -index; HF, heart failure. Note: Demographic data from this cohort have been partially reported in previous publications by our research group [14, 15, 21]. These data are reproduced here to provide context for the present original analysis of myocardial strain using Caas MR.

### 3.2 Strain Values for Healthy Volunteers Versus Heart Failure Patients

**Fig. 2.**
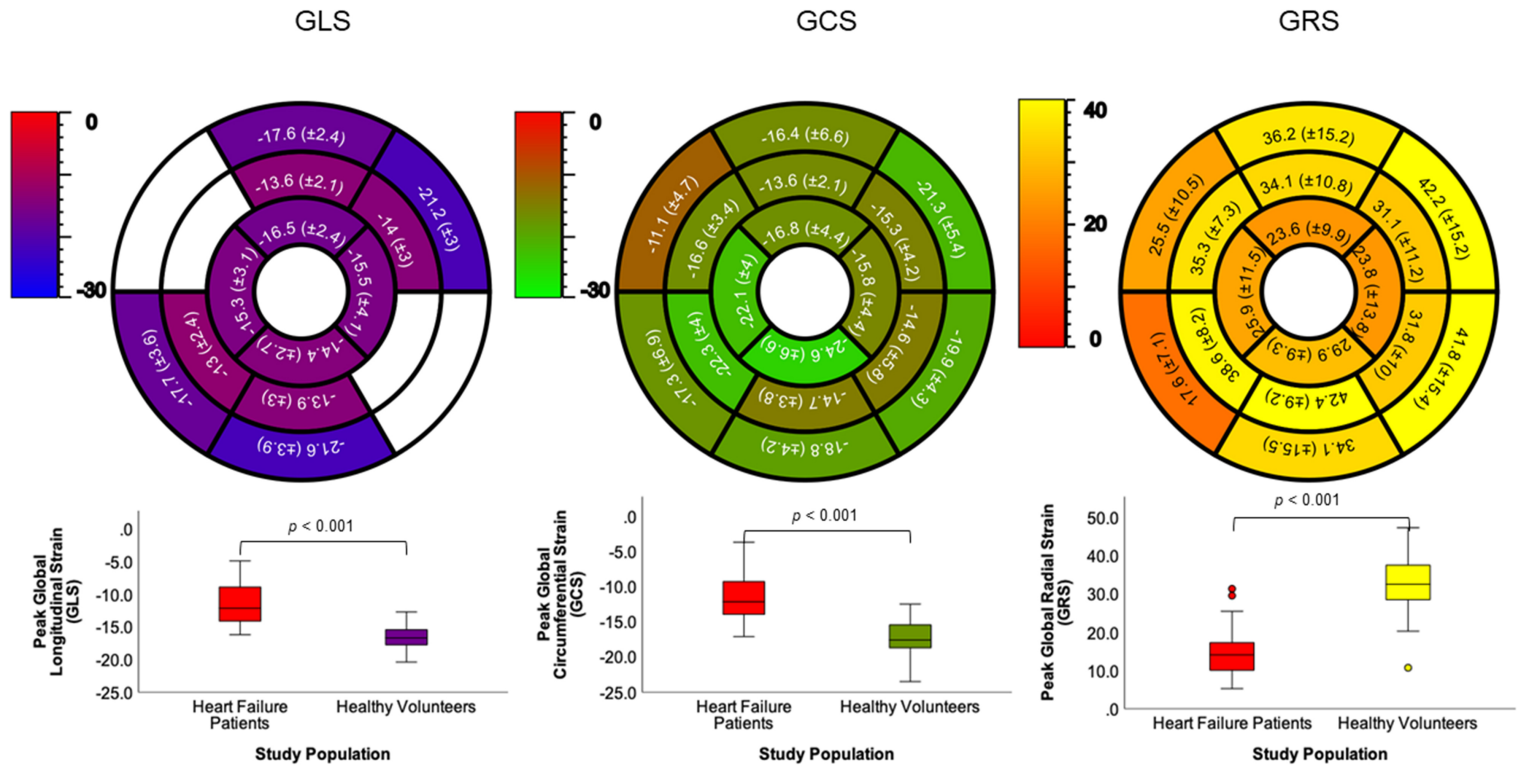
**Normal values for healthy volunteers**. Global and segmental normal values for GLS (left), GCS (middle) and GRS (right). Boxplots below show significant differences between healthy volunteers and HF patients; level *p* < 0.05. Values are mean ± SD (range). GLS, global longitudinal strain; GCS, global circumferential strain; GRS, global radial strain.

Mean GLS by Caas MR Strain was –15.8 ± 1.9% for healthy volunteers (absolute range –19.3 to –12.7) versus –11.7 ± 3.0% for HF patients (range –16.2 to –4.9; U = 127.50, *p* < 0.001), resulting in a normal value range (healthy volunteers mean ± 2.576 SD) of –17.0 to *–*14.6 for GLS. Mean GCS was –17.0 ± 2.6% for healthy volunteers (absolute range –23.5 to –12.8) versus –11.4 ± 3.3% for HF patients (absolute range –17.0 to –3.7; *p* < 0.001), resulting in a normal value range of –18.7 to –15.3 for GCS. Mean GRS was 27.3 ± 6.2% for healthy volunteers (absolute range 10.7 to 36.9) versus 14.5 ± 5.5% for HF patients (absolute range 5.2 to 31.4; U = 984.50, *p* < 0.001), resulting in a normal value range of 23.1 to 31.4 for GRS. Segmental values for healthy volunteers for GLS, GCS and GRS are presented in Fig. 2. A simplified approach for clinical use is proposed in Table 2.

**Table 2.** **Caas MR Strain–simplified LV myocardial strain values for clinical practice**.

	Healthy (normal strain)	Heart failure (impaired strain)
GLS (Caas MR Strain)	≤–15%	>–15%
GCS (Caas MR Strain)	≤–15%	>–15%
GRS (Caas MR Strain)	≥23%	<23%

LV, left ventricle; GLS, global longitudinal strain; GCS, global circumferential strain; GRS, global radial strain; Caas MR Strain, Pie Medical Imaging, Maastricht.

### 3.3 Strain Values Among Heart Failure Entities

**Fig. 3.**
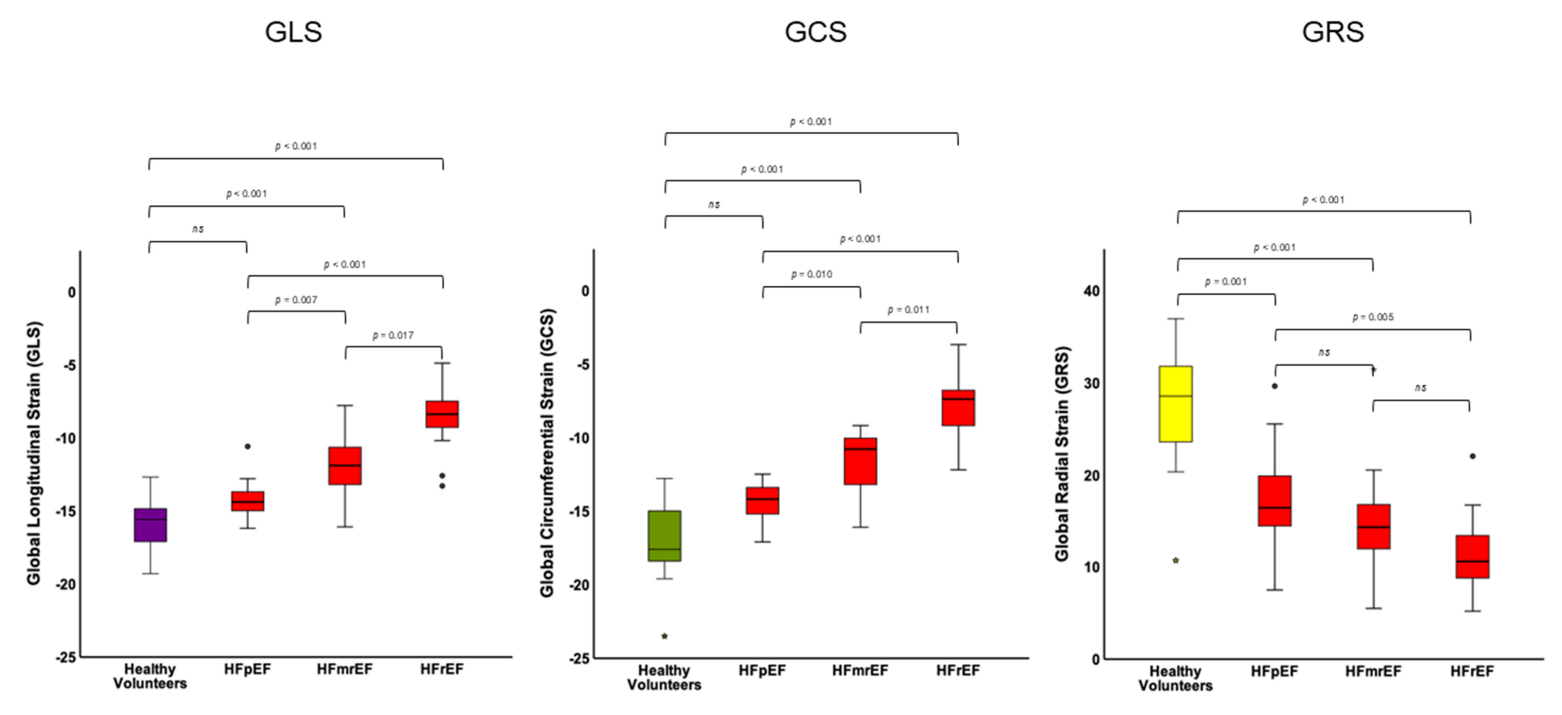
**Strain values for HF entities volunteers**. Global values for GLS (left), GCS (middle) and GRS (right) for HFpEF, HFmrEF and HFrEF patients and healthy volunteers. Significance level *p* < 0.05, ns, non-significant. HFpEF, HF with preserved ejection fraction; HFmrEF, HF with mid-range ejection fraction; HFrEF, HF with reduced ejection fraction; GLS, global longitudinal strain; GCS, global circumferential strain; GRS, global radial strain.

There was a statistically significant difference for GLS among HF patients with different HF entities, *p* < 0.001. Mean GLS by Caas MR Strain was (–14.3 ± 1.3) % for HFpEF patients (range –16.2 to –10.6) versus (–11.8 ± 2.2) % for HFmEF patients (range –16.1 to –7.8) versus (–8.5 ± 2.2) % for HFrEF patients (range –13.3 to –4.9). There was also a statistically significant difference for GCS among HF patients with different HF entities, H (2) = 36.18, *p* < 0.001. Mean GCS by Caas MR Strain was –14.4 ± 1.4% for HFpEF patients (range –17.1 to –12.5) versus –11.7 ± 2.0% for HFmEF patients (range –16.1 to –9.2) versus –7.8 ± 2.4% for HFrEF patients (range –12.2 to –3.7). There was also a statistically significant difference for GRS among HF patients with different HF entities, H (2) = 12.29, *p* < 0.001. Mean GRS by Caas MR Strain was 17.0 ± 5.5% for HFpEF patients (range 7.5 to 29.6) versus 14.9 ± 5.3% for HFmrEF patients (range 5.5 to 31.4) versus 11.2 ± 4.2% for HFrEF patients (range 5.2 to 22). Pairwise comparisons (Mann-Whitney U test) for GLS, GCS and GRS among different HF entities and healthy volunteers are presented in Fig. 3.

### 3.4 Inter-Rater Reliability of Strain Values

A high level of agreement was found when all participants were analyzed by two experienced readers (both EACVI level III) to assess inter-rater reliability of strain values. The average measure ICC for GLS by Caas MR Strain was 0.991 with a 95% CI from 0.986 to 0.994 (F = 110.319, *p* < 0.001). The average measure ICC for GCS by Caas MR Strain was 0.974 (95% CI 0.958 to 0.984, F = 38.310, *p* < 0.001) and the average measure ICC for GRS by Caas MR Strain was 0.980 (95% CI 0.969 to 0.988, F = 50.937, *p* < 0.001).

### 3.5 Inter-Vendor Comparison of Strain Values

**Fig. 4.**
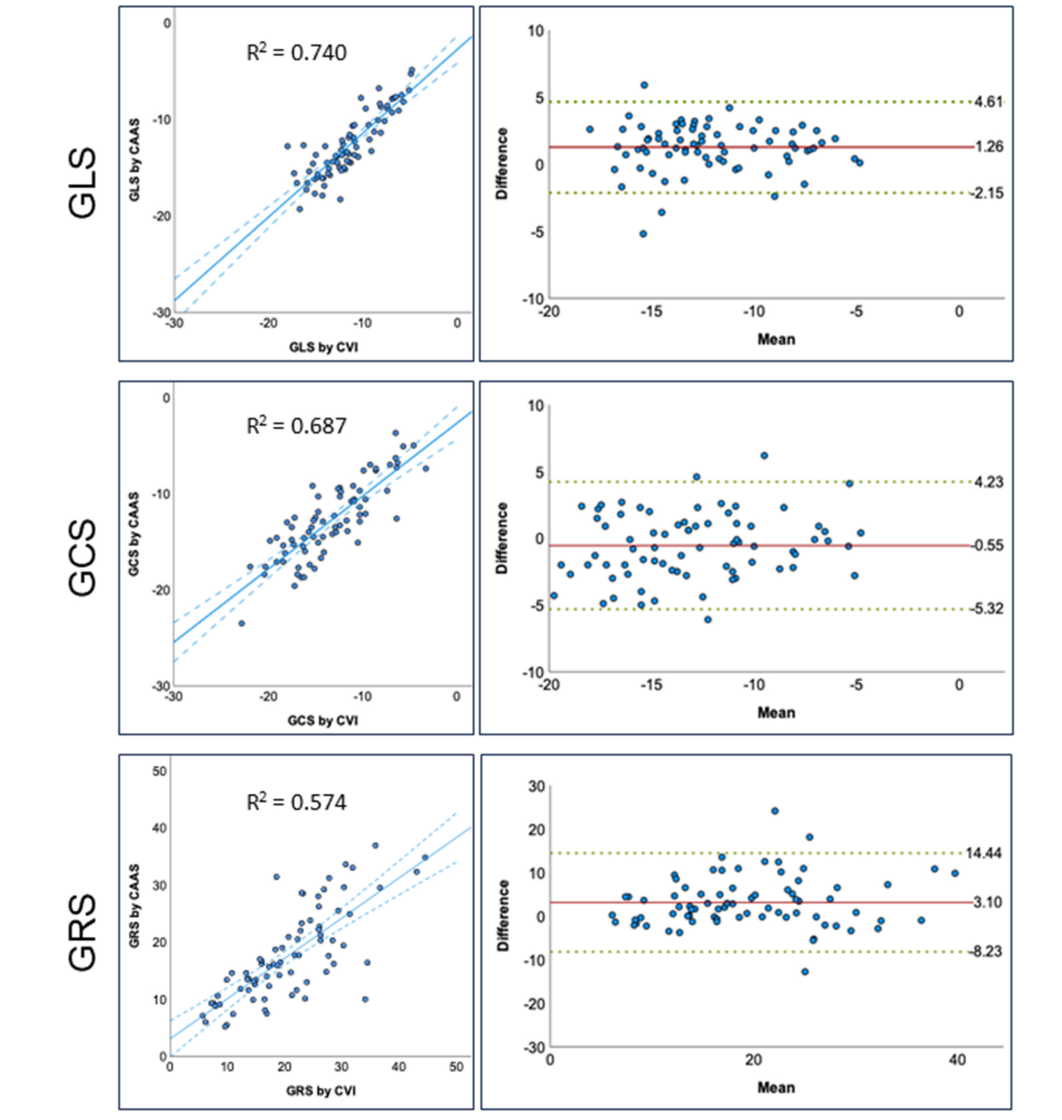
**Inter-vendor comparison for GLS, GCS and GRS**. Scatterplots and Bland-Altman analysis comparing Caas Strain MR with CVI42. GLS, global longitudinal strain; GCS, global circumferential strain; GRS, global radial strain; Caas MR Strain, Pie Medical Imaging, Maastricht, The Netherlands; CVI42, Circle Cardiovascular Imaging Inc., Calgary, Canada.

Vendor-specific strain values for healthy volunteers and HF patients are presented in Table 3. After careful evaluation of any systemic bias or outliers in the data using Bland-Altman plots, computation of the Pearson correlation coefficient showed a significant positive correlation for GLS for Caas MR Strain versus CVI42 [*r* = 0.86, *p* < 0.001]. Similarly, a significant positive correlation was found for GCS for Caas MR Strain versus CVI42 [*r*(73) = 0.83, *p* < 0.001], as well as GRS for Caas MR Strain versus CVI42 [*r*(73) = 0.76, *p* < 0.001]. Bland-Altman plots and scatterplots for GLS, GCS and GRS for all three vendors are shown in Fig. 4.

**Table 3.** **Vendor-specific LV strain values for healthy volunteers and heart failure patients**.

	Volunteers (n = 19)	HFpEF (n = 19)	HFmrEF (n = 20)	HFrEF (n = 17)
GLS (Caas MR Strain)	–15.8 ± 1.9 (–19.3 to –12.7)	–14.3 ± 1.3 (–16.2 to –10.6)	–11.8 ± 2.2 * (–16.1 to –7.8)	–8.5 ± 2.2 * (–13.3 to –4.9)
GLS (CVI4)	–14.9 ± 1.9 (–18 to –11.7)	–12.8 ± 1.4 (–15.1 to –10.9)	–10.5 ± 2.0 * (–14.3 to –6.2)	–7.2 ± 1.8 * (–12.1 to –4.8)
GCS (Caas MR Strain)	–17.0 ± 2.6 (–23.5 to –12.8)	–14.4 ± 1.4 (–17.1 to –12.5)	–11.7 ± 2.0 * (–16.1 to –9.2)	–7.8 ± 2.4 * (–12.2 to –3.7)
GCS (CVI4)	–17.4 ± 2.5 (–22.8 to –14.2)	–15.9 ± 2.3 (–19.8 to –11.7)	–11.7 ± 2.0 * (–15.3 to –6.4)	–8 ± 2.7 * (–14.7 to –3.3)
GRS (Caas MR Strain)	27.3 ± 6.2 (10.7 to 36.9)	17.0 ± 5.5 * (7.5 to 29.6)	14.9 ± 5.3 * (5.5 to 31.4)	11.2 ± 4.2 * (5.2 to 22.0)
GRS (CVI4)	28.9 ± 6.9 (20.7 to 44.6)	25.4 ± 5.5 (16.6 to 34.5)	17.2 ± 3.3 * (9.9 to 23.6)	11 ± 4.3 * (5.7 to 22.8)

Values are mean ± SD (range). LV, left ventricle; HFpEF, heart failure with preserved ejection fraction; HFmrEF, heart failure with mid-range ejection fraction; HFrEF, heart failure with reduced ejection fraction; GLS, global longitudinal strain; GCS, global circumferential strain; GRS, global radial strain; Caas, strain values obtained with Caas MR Strain software; CVI42, strain values obtained with CVI42 software. Caas MR Strain, Pie Medical Imaging, Maastricht, The Netherlands; CVI42, Circle Cardiovascular Imaging Inc., Calgary, Canada. * Comparisons between HF subgroups and healthy volunteers are statistically significant (*p* < 0.05) for CVI42 and Caas-derived strain values (Dunn’s test with Bonferroni correction). Kruskal–Wallis test results (χ^2^(df)) are reported in the table; CVI42 pairwise comparisons use adjusted *p*-values as indicated by the asterisk.

## 4. Discussion

To the best of our knowledge, this study is among the first to demonstrate that calculation of GLS using only two long axis (LAX) acquisitions, 2CH and 4CH, is feasible and shows very good agreement with CVI42-based GLS assessment (*r* = 0.86, *p* < 0.001). Strong correlations were observed between Caas MR Strain and CVI42 for GCS (*r* = 0.83, *p* < 0.001) and GRS (*r* = 0.76, *p* < 0.001), supporting the robustness of this software across multiple strain components. Our study thus highlights reliable comparability in regards to Circle’s CVI42, which in turn has been validated against other FT strain solutions [12]. This finding could prove beneficial to allow for better comparison of results from different sites and could potentially improve patient follow-up [18, 19].

Our findings suggest that FT-MRI strain analysis using Caas MR Strain may help differentiate between healthy individuals and patients with HF. In this study, we were able to propose cutoff values for GLS above –14.6%, for GCS above –15.3% and GRS below 23.1% to define pathological findings. Given the higher SD for GRS, the values seem less reliable. This is in line with previous findings suggesting increased reproducibility for GLS and GCS but less for global radial strain measurements [19, 25, 26].

A recent study from our research group identified a GLS cut-off point (≥–15%) that showed potential for distinguishing HF patients from healthy controls using CVI42-based strain analysis [27]. Our results using CAAS MR Strain suggest a similar threshold. Furthermore, the method allows for some discrimination regarding the underlying HF subgroups. However, we were not able to show a clear discriminatory power for GLS, GCS, and GRS to differentiate between HFpEF, HFmrEF, and HFrEF. Similarly, we were not able to show the widely accepted significant decrease in GLS and GCS in HFpEF patients as compared to healthy adults, however, a trend towards decreased strain even in HFpEF patients is clearly visible in our cohort. This is possibly due to the relatively low number of patients in this study and somewhat contradictory to established diagnostic algorithms for HFpEF using different imaging modalities [2, 15, 28].

While in our study, the classification of HF was conducted based on an imaging perspective according to LVEF and FT-MRI strain parameters, the classified categories can generally be defined as different stages or severity of clinical HF, such as the classification systems used by the New York Heart Association (NYHA). In future studies, the combination of strain analysis with established clinical classifications may improve phenotyping and aid clinical decision-making.

The lower GLS observed in our study, compared to the literature, likely reflects both physiological and methodological factors. First, our control group was relatively older (mean age 63 ± 9 years). Previous CMR-FT studies have reported age-related variations, with progressive reductions in GLS with increased age [13, 29]. Second, gender stratification was limited in our cohort. Prior work has shown that females exhibit higher (more negative) GLS than males, with differences of approximately 1–2% [26, 29]. Since our group was composed of a balanced mix of both sexes, the overall values would be expected to be lower than those reported in female-only or sex-stratified cohorts. Third, strain was quantified using Caas MR Strain, which applies block-matching and Lagrangian strain computation. Inter-vendor comparisons have demonstrated systematic differences of 2–3% for GLS between commonly used platforms, mainly attributable to differences in contour definition, Region of Interest (ROI) tracking, and algorithm implementation [18, 19]. Therefore, age dependency, gender effects, and vendor-related variability could contribute to a composite deviation of 4–6% relative to reference values. This aligns with the data observed in our cohort and strongly suggests that the differences are methodological rather than pathological.

In patients with advanced HF, myocardial thinning and impaired wall motion may reduce the accuracy of endocardial and epicardial border definition, which could decrease the accuracy of FT-MRI strain measurements. While previous studies have shown good agreement between FT and reference techniques (tagging or Strain-Encoded (SENC)) [12, 19], strain accuracy can be reduced as ventricular remodeling increases [25, 26]. In our study, contours were carefully reviewed and manually corrected by two experienced readers (EACVI level III), but this variability remains an inherent limitation of FT-based analysis in abnormal myocardium.

A strength of our study is the reproducibility of myocardial strain assessment. The excellent inter-rater ICC values observed for GLS, GCS, and GRS reflect low ROI-dependent variability, thereby providing quantitative error estimates for accuracy-dependent changes. This methodological robustness further supports the assumption of a high reliability of strain values measured by this novel tool.

Our study only reports values for 1.5 T and for images acquired with the use of acceleration technique SENSE, which is widely adopted in clinical practice. Therefore, although our results are specific to 1.5 T scanners and acquisitions using SENSE acceleration, they may still be cautiously extrapolated to other settings, as previous studies have suggested minimal influence of field strength and acceleration techniques on strain values [19]. Since strain rate has been shown to be of incremental value in HF patients, future analysis should ideally include these time-dependent parameters [14]. Future directions of strain analysis hold the potential for including prospective cohorts for prognostic evaluation and assessing large populations using artificial intelligence [30, 31].

Segmental strain analysis was also performed and is shown in Fig. 2. Although our primary objective focused on global strain values, segmental strain may provide additional diagnostic information. However, segmental strain is generally associated with higher variability and lower reproducibility, which limits its immediate applicability in clinical practice. Future studies with larger cohorts should explore the diagnostic potential of segmental strain values in different HF populations.

This study has several limitations. We did not report age or gender-specific values, which could be beneficial since it has been shown that age is a potential confounder and younger patients are reported to have more pronounced myocardial deformation values [13, 29]. However, since we chose to study a group of HF patients, our values from relatively old volunteers (mean age 63 ± 9) are perhaps relevant to identify truly abnormal values among typical cardiac patients. A larger study population would allow reporting of age-specific normal values. The literature provides somewhat contradictory findings regarding gender, with most studies finding differences in strain values according to gender, although no uniform consensus is reported [13, 19, 26, 29]. The time required for analysis with both platforms was not systematically recorded; however, based on the authors’ experience, both tools provide semi-automated workflows with comparable post-processing time. Finally, the healthy control group was not age-matched to the HF cohort. This was due to the challenge of including older controls without cardiovascular comorbidities or subclinical abnormalities.

## 5. Conclusion

CMR imaging assessment of left ventricular myocardial strain using Caas MR Strain software reliably identifies HF patients. Discrimination between HF subgroups by GLS, GCS, and GRS showed statistically significant trends. However, given the limited sample size, these findings should be considered exploratory and require confirmation in larger studies. Inter-vendor agreement was most robust for GLS and GCS, but less robust for GRS. For practical clinical use, we propose cut-off values for GLS above –15%, GCS above –15%, and GRS below 23% to better define pathological changes.

## Consent for Publication

Patients signed informed consent regarding publishing their data.

## Availability of Data and Materials

All data will be available from the corresponding author upon reasonable request.
